# A comparison of automated anatomical–behavioural mapping methods in a rodent model of stroke^☆^

**DOI:** 10.1016/j.jneumeth.2013.05.009

**Published:** 2013-09-15

**Authors:** William R. Crum, Vincent P. Giampietro, Edward J. Smith, Natalia Gorenkova, R. Paul Stroemer, Michel Modo

**Affiliations:** a King's College London, Institute of Psychiatry, Department of Neuroimaging, London SE5 8AF, UK; b King's College London, Institute of Psychiatry, Department of Neuroscience, London SE5 9NU, UK; c ReNeuron Ltd., Guildford GU2 7AF, UK; d Department of Radiology, McGowan Center for Regenerative Medicine, University of Pittsburgh, Pittsburgh, PA, USA

**Keywords:** Voxel lesion symptom mapping, Tensor-based morphometry, Stroke, Magnetic resonance imaging, Image registration

## Abstract

Neurological damage, due to conditions such as stroke, results in a complex pattern of structural changes and significant behavioural dysfunctions; the automated analysis of magnetic resonance imaging (MRI) and discovery of structural–behavioural correlates associated with these disorders remains challenging. Voxel lesion symptom mapping (VLSM) has been used to associate behaviour with lesion location in MRI, but this analysis requires the definition of lesion masks on each subject and does not exploit the rich structural information in the images. Tensor-based morphometry (TBM) has been used to perform voxel-wise structural analyses over the entire brain; however, a combination of lesion hyper-intensities and subtle structural remodelling away from the lesion might confound the interpretation of TBM. In this study, we compared and contrasted these techniques in a rodent model of stroke (*n* = 58) to assess the efficacy of these techniques in a challenging pre-clinical application. The results from the automated techniques were compared using manually derived region-of-interest measures of the lesion, cortex, striatum, ventricle and hippocampus, and considered against model power calculations. The automated TBM techniques successfully detect both lesion and non-lesion effects, consistent with manual measurements. These techniques do not require manual segmentation to the same extent as VLSM and should be considered part of the toolkit for the unbiased analysis of pre-clinical imaging-based studies.

## Highlights

• The first application of voxel lesion symptom mapping (VLSM) in rodents.
• A comparison of VLSM with tensor based morphometry (TBM) methods in a stroke model.
• Comparison of automated techniques with manual measurements and model power calculations.
• Analysis of both local and non-local lesion effects.
• Correlation of structural change with behaviour.

## 1 Introduction

Stroke remains the main cause of severe adult disability in the Western world. However, little is known about the relationship between the neuro-anatomical damage caused by stroke and the resulting behavioural deficits. The localisation of functional deficits has predominantly focussed on the use of functional magnetic resonance imaging (fMRI) (Cirstea et al., 2011; Dijkhuizen et al., 2003; Rehme et al., 2011; Weber et al., 2008), which indicates changes in functional networks that typically support simple behaviours, such as arm movement (Rehme et al., 2011). However, the use of fMRI to investigate functional responses in the peri-lesion area is based on the assumption that changes in neurovascular coupling and the potential vascular response are akin to the changes observed in the normal brain. There is ample evidence that this assumption is not the case (Beck and Plate, 2009; Bonakdarpour et al., 2007; Hughes et al., 2010), and therefore, a structural–behavioural analysis would be more informative (Alexander et al., 2010; Ashioti et al., 2007; Modo et al., 2009). However, stroke offers a particularly challenging combination of structural and behavioural features for analysis as follows: (i) primary focal lesions, (ii) secondary structural (remodelling) effects, (iii) a spectrum of behavioural effects, and (iv) a chronic time-course of structural and behavioural effects. Acute stroke results in localised hyper- or hypo-intensities in MRI directly associated with the lesion. As these effects do not correspond with normal anatomy, localised hyper- or hypo-intensities in MRI present particular challenges for automated analysis based on image registration (Crum et al., 2004); thus, many studies have resorted to using manual segmentation methods. Other stroke effects include changes in morphology and tissue density away from the original lesion site, which might develop over time in a subtle or diffuse fashion. These features can be successfully reproduced in a rodent model, facilitating the detailed examination of structural–behavioural interactions under controlled conditions.

In principle, automated registration-based methods, such as voxel based morphometry (VBM) (analysing tissue intensities) and tensor-based morphometry (TBM) (analysing local shape/volume differences), detect changes in tissue density (or lesion-related pathological changes) that, in turn, might be correlated with measures of behavioural impairments (Kim et al., 2007). However, the efficacy of VBM for lesion detection is uncertain (Mehta et al., 2003) and as in humans, accurate brain tissue classification in rodents, in the presence of lesions, is challenging. Conversely, TBM, is difficult to interpret in an environment that includes structure- and intensity-modifying lesions, as well as pure morphological changes. Thus, Voxel-Lesion-Symptom-Mapping (VLSM) is an alternative technique, which focuses on lesion-behaviour correlations (Bates et al., 2003; Dronkers et al., 2004); however, this method requires stroke-lesion segmentation and is only applicable in brain regions where at least some subjects have lesion voxels defined. The VLSM approach has previously been applied in patients with stroke damage to identify localised regions which correlate with observed behavioural impairments, such as motor coordination (Kalenine et al., 2010; Konczak et al., 2010; Lo et al., 2010). In these studies, the extent of the stroke lesion is established individually in each subject and subsequently used to define lesion and non-lesion subject groups for testing *at each voxel*. Applying a similar approach to animal studies would establish the brain regions involved in behavioural impairments and enable a more detailed neuro-pathological investigation of the cellular and molecular aspects underlying these morphological changes. However, as previously stated, the location and appearance of stroke is highly variable, even in models. In addition, using a subjective binary definition of the lesion presence at each voxel does not account for intensity variations observed within the lesion environment, which might encode more subtle correlations. The currently available techniques for automatic lesion identification in individual scans (Shen et al., 2008) are not sufficiently reliable or robust to be deployed routinely in this setting, further limiting scalability to larger studies. Nevertheless, VLSM approaches are resistant to confounding factors from non-lesion effects.

In many ways, VLSM and TBM are complementary. VLSM does not exploit the rich structural information available in whole brain MRI, while TBM does not exploit the morphological information available from expert delineation of stroke lesions. However, behavioural measures can be correlated with the structural volumetric differences obtained by TBM at each voxel. Differences in the volumetric effects might be directly associated with the lesion, or more subtle and distal to the lesion. Regardless of the analysis techniques employed, an over-arching concern is that animal studies are limited in size through practical and ethical considerations that critically limit the power available for voxel-wise statistical analysis. Therefore, while there are sophisticated tools available for the analysis of brain imaging studies, the efficacy of these methods in challenging pre-clinical studies applications warrants further investigation. In this study, we applied VLSM, and TBM in an imaging and behavioural study of a rat stroke model. Manual segmentation of both lesion and anatomy is used to validate the automated methods. A power analysis of exemplar experiments is performed to estimate effect sizes.

## 2 Methods

Behavioural data and MR images are available upon request from the authors.

### 2.1 Stroke model

Sprague-Dawley rats (Harlan, UK) between 280 and 330 g in body weight were acclimatised for at least a week prior to surgery and randomly allocated to sham (*n* = 16) or stroke (*n* = 42) groups (Smith et al., 2012). The animals were maintained on a 12 h-light:12 h-dark schedule with food and water available *ad libitum*. All procedures were performed in accordance with the UK Animals (Scientific) Procedures Act 1986 and the ethical review process of King's College London. The stroke group underwent Middle Cerebral Artery Occlusion (MCAo), as previously described (Modo et al., 2000b). The sham group underwent similar filament insertion without occlusion; thus, any structural/behavioural effects associated with surgery alone were present in both groups.

### 2.2 Imaging

^1^H MRI was performed at 10 days following MCAo using a horizontal-bore 7 T MRI scanner (Varian, USA) with a custom-made 63-mm internal diameter radiofrequency coil (David Herlihy, Imperial College). The animals were anaesthetised for the duration of the imaging session using isoflurane (4% induction and 2% maintenance) in a mixture of O_2_:N_2_O (30:70). The MR image acquisition consisted of a fast spin echo sequence (TR = 3000 ms, ETL = 4, ESP = 15, Effective TE = 60 ms, kzero = 4, averages = 10, matrix size = 128 × 128, FOV = 3 cm × 3 cm, in plane resolution = 0.234 mm × 0.234 mm, number of slices = 45, slice thickness = 0.5 mm, and total scanning time = 16 min).

### 2.3 Manual image analysis

**Fig. 1:**
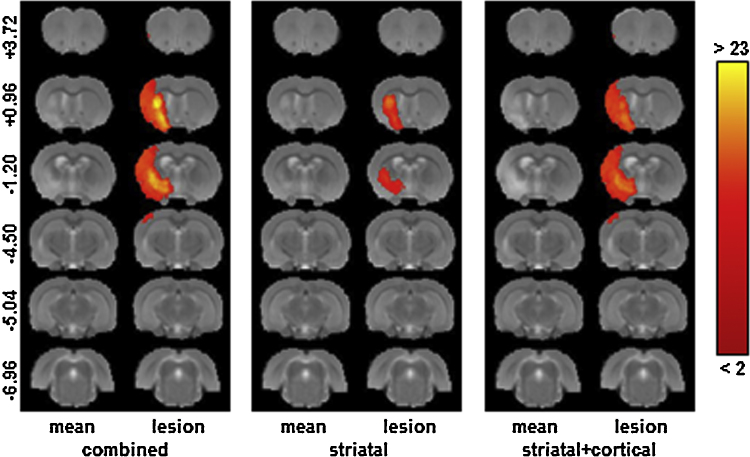
The distribution of lesion voxels in the stroke model. The mean MR image is shown together with a colour overlay indicating the number of subjects with a lesion defined at each voxel for (i) the entire stroke group, (ii) subjects with predominantly striatal lesions and (iii) subjects with predominantly striatal and cortical lesions. This figure was generated using registered data, but the lesion outlines were drawn on the original unregistered scans.

To quantify the stroke volume and define lesion masks, a region of interest (ROI) was drawn across all slices showing hyper-intensity within the grey matter (defined as more than one standard deviation above the mean of a homologous area in the unaffected contralateral hemisphere) (Ashioti et al., 2007). Fig. 1 shows the distribution of the resulting lesion voxels for (i) the entire lesioned cohort, and for two sub-groups detected during the mask generation, (ii) the striatal lesion only group and (iii) the striatal and cortical lesion group. The ROIs for four anatomical structures (striatum, cortex, hippocampus and ventricles) were defined using a rat brain atlas (Paxinos and Watson, 2007) and manually drawn on each scan; the ipsilateral and contralateral volumes were measured for each structure. All ROI analyses were conducted using Jim 5.0 software (Xinapse Systems Limited, Aldwincle, UK). In all cases, intra- and inter-observer reproducibility training was performed to ensure a segmentation consistency of at least 95% for each structure.

### 2.4 Behavioural assessment

*Grip strength*. To investigate forelimb motor dysfunction, we used a grip strength meter (GSM; TSE Systems, Germany), as previously described (Vernon et al., 2010). The grip strength (gs) was recorded for left (gsl) and right (gsr) forepaws.

*Foot-fault*. The foot-fault (ff) test (Stroemer et al., 1998) measures the ability to integrate motor responses. The rats were placed onto a suspended mesh wire (40 cm × 150 cm, 50 cm height and, 5 cm mesh size) and the correct and incorrect placements of the left (ffl) and right (ffr) forelimb were recorded over four 60-s trials.

### 2.5 Pre-processing for automated analysis

**Fig. 2:**
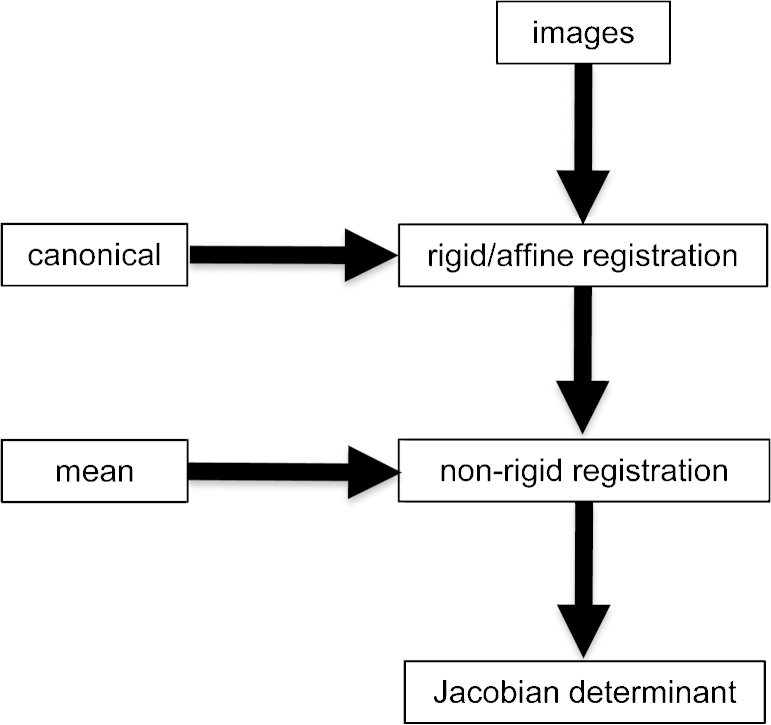
Schematic of the image pre-processing pipeline. (a) Rigid registration of all images to a canonical reference. (b) Non-rigid registration of all images to the mean sham image. (c) Generation of maps of Jacobian determinants encoding apparent fractional volume difference between each image and the sham mean.

All images were pre-processed using the following procedure (Fig. 2). The general strategy for all methods was to first use global (rigid) registration to remove positioning differences and generate group mean images, followed by local (non rigid) registration between each scan and a reference (the sham mean image). The non-rigid registration generates maps of apparent volume difference between each scan and the reference; these maps are subsequently subjected to TBM analysis.

#### 2.5.1 Global registration

**Fig. 3:**
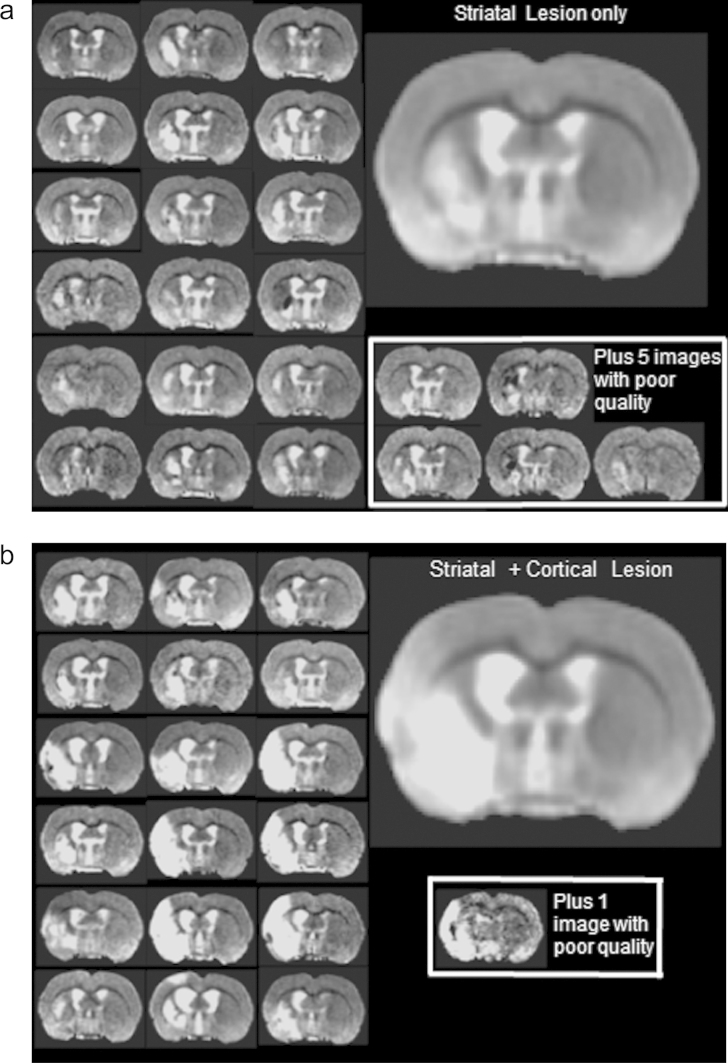
Lesion variation and structural differences in the lesion sub-groups. A representative slices from each member of (a) the striatal lesion and (b) the striatal + cortical lesion groups are shown together with the mean image for each group. The poor-quality images are shown separately and excluded from further analysis. The image contrast was adjusted to emphasise the hyper-intensities.

We used the FLIRT image registration software (Jenkinson et al., 2002; Jenkinson and Smith, 2001) applied over the entire image cohort (Crum et al., 2013). A single sham animal with a good quality scan (minimal artefacts, centrally positioned and high SNR) was selected as a canonical reference. A brain ROI on the reference was obtained manually using MRIcron (Rorden et al., 2007). Each animal was registered to the reference using six degrees-of-freedom (dof) to correct for positioning differences. The image and registration quality was assessed using careful visual inspection. Images with poor signal-to-noise ratios or imaging artefacts, or images poorly registered to the reference were excluded from further study, resulting in the removal of two sham and six stroke images. We corrected for low-frequency -intensity variations using N3 (Sled et al., 1998). The mean registered images of each group were constructed for visualisation, and the mean registered sham image was adopted as a reference for the local registration step. Fig. 3 shows the variation in lesion location and image quality across the cohort. Animals with damage to the striatum (Fig. 3a) can be visually distinguished from animals with a striatal + cortical lesion (Fig. 3b).

#### 2.5.2 Local registration

Each globally registered scan was subsequently registered to the mean sham image using a high-dimensional diffeomorphic (topology preserving) “fluid” registration technique (Crum et al., 2001, 2005; Freeborough and Fox, 1998). This registration corrects pure morphological (shape) differences through local warping. Hyper-intense regions associated with the stroke lesion are interpreted as topological differences mapped onto images without a corresponding lesion. These differences manifest as extremes of apparent local volume differences between scans where the registration reduces the lesion volume (through local warping) to match the normal tissue. Therefore, we expect to capture both localised lesion-related effects and less extreme, but distal, structural remodelling effects. The fluid registration is parameterised (regularised) using two “viscosity” parameters, *μ* and *λ*, set to normalised standard values of 1 and 0, respectively (Christensen et al., 1996), which balance the effects of voxel displacement versus voxel volume change and represent an unbiased parameter selection. In the Appendix, we investigate the effect of changing this balance. The overall structural differences between scans are encoded in the displacement fields representing the mapping from each image to the mean sham; these fields are converted into Jacobian determinant maps of localised fractional volume change for subsequent analysis.

### 2.6 Automated image analysis

The following techniques were applied:

#### 2.6.1 Voxel lesion symptom mapping (VLSM)

We implemented VLSM using a previously described method (Bates et al., 2003; Kimberg, 2009).^1^ Briefly, VLSM involves a voxel-by-voxel analysis to determine whether groups, defined on the basis of lesion presence (or absence), at each voxel differ significantly in recorded behaviour. Notably, the contribution of the image data to this analysis is limited to providing lesion-masks for each animal and a neuro-anatomical coordinate system in which to perform the analysis. This analysis does not incorporate the voxel intensity, volume or shape information derived from the images. Differences in the four behavioural measures associated with manual lesion-assignment at each voxel are evaluated.

#### 2.6.2 Tensor-based morphometry (TBM)

A statistical analysis of the log-Jacobian determinant maps was performed to compare the local apparent volume changes between the stroke and control groups (Ashburner et al., 1998).

(i) Voxel-wise analysis of Jacobian determinant maps identifies regions that differ significantly between the sham and lesion groups. Note that this analysis does not incorporate behaviour.
(ii) The voxel-wise correlation between behaviour and apparent local volume difference with respect to the template was evaluated in a single group.

### 2.7 Statistical analyses

#### 2.7.1 Behaviour and MRI manual volume comparisons

An a priori power analysis was performed using G*Power 3 (University of Trier) to ensure at least 80% power (1 − *β*) with an estimated effect size *f* of 0.2 for the foot-fault test (the primary outcome measure). All other statistical analyses were conducted using SPSS20 for the Mac (IBM), and the graphs were generated using Prism 5 software (GraphPad). Differences in the behaviour and MRI volumes for each group (Control and MCAo) and hemisphere (ipsi- and contralateral) were analysed using a two-way analysis of variance (ANOVA) followed by the Bonferroni post hoc test to correct for multiple comparisons. Differences between different groups (Control, Combined, Striatal, Striatal + Cortical) were established using a one-way ANOVA followed by Bonferroni post hoc tests. *p* < 0.05 was considered significant.

#### 2.7.2 Behaviour and MRI manual volume correlations

The correlation analyses between behaviour and MRI were conducted with SPSS20 for the Mac using the Pearson product moment correlation coefficient. Bonferroni corrections were applied to account for multiple comparisons. Correlations from 0 to 0.3 were considered weak, from 0.3 to 0.7 were considered medium, and from 0.7 to 1 were considered strong. The sample size used here had sufficient power (80%) to detect medium correlations at the *p* < 0.05 significance level.

#### 2.7.3 Automated techniques

Automated techniques were applied to the sham and three lesion sub-groups and the four behavioural measures as appropriate.

##### 2.7.3.1 VLSM statistics

Unequal-variance two-tailed *t*-tests at each voxel (to assess differences in the four behavioural scores) were computed using non-parametric permutation testing (Bullmore et al., 1999) to determine significance. Briefly, the group membership (i.e., “sham” or “stroke”) is repeatedly and randomly permuted, and the *t*-statistic is recomputed at each permutation to generate the null distribution of the statistic. The significance level is defined as the probability that the original *t*-statistic could have been obtained by chance from the null distribution. The False discovery rate (Genovese et al., 2002) with *q* = 0.05 was used for multiple comparison correction. Unlike standard cohort-based analyses such as TBM (where group size and membership is based on cohort label alone and therefore identical at each voxel), the group membership, size and, therefore, available statistical power varied from voxel to voxel. A cut-off for minimum group-size must be specified; in this work, we set the minimum group-size for the VLSM analysis to five.

##### 2.7.3.2 TBM statistics

As with VLSM, a two-tailed *t*-statistic, assuming unequal variance between groups, was computed at each voxel in the brain, generating approximately 42,000 tests. In practice, we increased the effective number of permutations at each voxel by pooling null-distribution statistics from other voxels in the mask to permit more accurate multiple comparison corrections. The minimum number of permutations required is approximately equal to the number of voxels/FDR significance level = 42,000/0.05 = 840,000 permutations (in this study). We calculated more than this minimum number of permutations for robustness and retain the option to threshold at higher significance levels.

For the analysis of behaviour, log-Jacobian determinant maps were correlated with the two behavioural measurements (*grip strength* and *foot-fault*). The non-parametric Spearman rank correlation coefficient, *ρ* (i.e., the Pearson product moment correlation coefficient, *r*, of the ranked variables) between the behavioural data and the log-Jacobian determinant was computed for each animal at each intra-cerebral voxel. The significance of *ρ* was determined using the permutation testing procedure, as described above for VLSM.

#### 2.7.4 Power analysis of image statistics

To establish the minimum likely detectable effect sizes at a voxel under standardised conditions, we conducted a model power-analysis of the three automated analysis techniques using the STPLAN software.^2^

VLSM: We modelled a two-group comparison with *n*1 = *n*2 = 15 animals, *p* = 0.05 and power = 0.8, corresponding to conditions where half the cohort is lesioned at a voxel and the remainder of the cohort is not. We based the observations on the foot-fault behavioural tasks, and arbitrarily assigned an unlesioned observation mean (standard deviation) of 20% (10%) (a relatively poor performance for the sham group) and a model lesion observation standard deviation of 10%. The resulting minimum effect size in the lesion group was calculated as 31%.

*TBM (i)*: We modelled a two-group lesion-sham volumetric comparison with *n*1 (lesion) = 30 and *n*2 (sham) = 10 using *p* = 0.05 and power = 0.80. The sham mean (standard deviation) Jacobian determinant was set to 1.0 (0.05) (i.e. no volume change with respect to the reference). The standard deviation of the lesion Jacobian determinant was set to (i) 0.05 and (ii) 0.20. The resulting minimum mean (log(mean)) (i.e., the effect size for the lesion group) was calculated as (i) 1.055 (0.054) and (ii) 1.115 (0.110), respectively.

*TBM (ii)*: We modelled behavioural correlation, with *p* = 0.05 and power = 0.8, in a single-group of *n* = 30 animals, generating a minimum detectable correlation of 0.485, as an alternative hypothesis.

## 3 Results

### 3.1 Manual image analysis

**Fig. 4:**
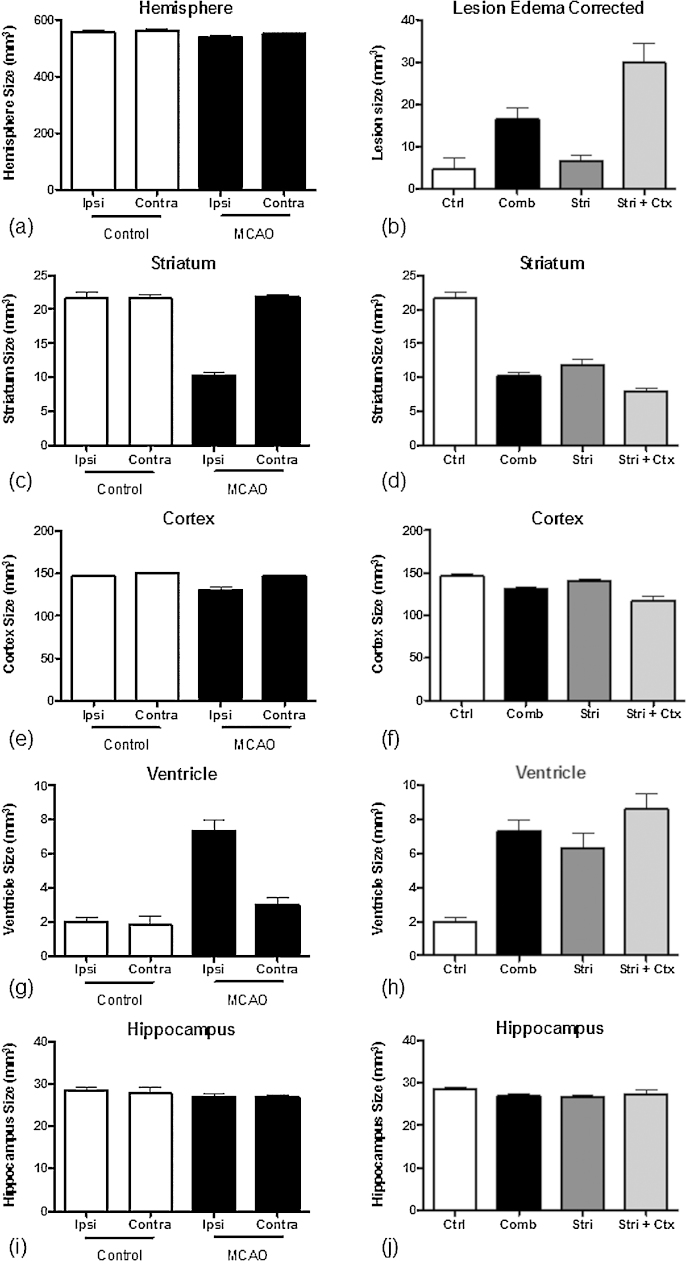
Manual region-of-interest analysis of stroke damage. (a) Brain hemisphere volume, (b) oedema-corrected lesion volume, (c and d) Striatum, (e and f) Cortex, (g and h) Ventricle, (i and j) Hippocampus. Ipsilateral and contralateral results for the sham and MCAo groups are shown in figures (a), (c), (e), (g) and (i). The results for the sub-groups with predominantly striatal and mixed striatal + cortical lesions are shown in figures (b), (d), (f), (h) and (j). See the text for further details.

There was no significant difference in the hemispheric brain volume between stroke and sham groups (Fig. 4a). The lesion volume (Fig. 4b), i.e., the region where cells were dying due to the occlusion, was significantly larger (*p* < 0.001) in the stroke group (approximately 20 mm^3^) compared with shams subjected only to the ligation of the common carotid (≤5 mm^3^). Although all animals underwent 60 min of occlusion, two-thirds of these animals exhibited striatal damage, with a lesion volume of approximately 10 mm^3^ while the remaining animals showed damage to both striatum and cortex, with a volume of 30 mm^3^ (significantly different volume with *p* < 0.001).

Due to the trajectory of the MCA, the striatum is the region most affected by occlusion. All animals with MCAo exhibited damage to this structure, effectively decreasing the striatal tissue to 50% of its original volume (Fig. 4c and d) (*p* < 0.001). Animals with striatal + cortex lesions showed significantly greater damage (as evidenced by volume reduction) to the striatum compared with animals with striatal-only lesions, likely reflecting differences in the vascular anatomy between these two cohorts of animals. For the cortical volumes, the same group differences were observed. The overall shrinkage of the ipsilateral cortex (Fig. 4e and f) reflected a significant decrease in the cortical volume in animals with both striatal and cortical damage (*p* < 0.05). Ventricular volume increases corresponding with cortical volume decreases were also observed (Fig. 4g and h). As expected, no change in the hippocampal volume was detected.

### 3.2 Behavioural assessment

**Fig. 5:**
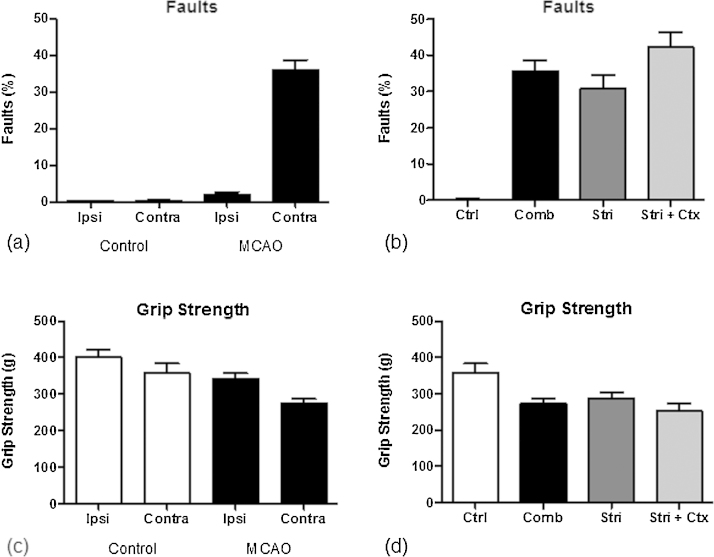
Behavioural testing results. (a) Foot-faults ipsilateral and contralateral to the sham-procedure or lesion, (b) foot-faults corresponding to the lesion sub-groups for the contralateral paw, (c) grip strength ipsilateral and contralateral to the sham-procedure or lesion, and (d) grip strength corresponding to the lesion sub-groups.

Behavioural impairment reflects the underlying anatomical damage and concomitant disruption of functional networks; impairments of motor function are commonly observed in stroke and were measured using the foot-fault (ff) and grip strength (gs) tasks. The animals in the stroke group showed a highly significant increase in ffl (i.e., l = left = contralateral to the lesion) (*p* < 0.001) and a smaller significant increase in ffr (i.e., *r* = right = ipsilateral to the lesion) (*p* < 0.05) (Fig. 5a). Animals with striatal + cortical lesions exhibited a more severe deficit in this task compared with animals with only striatal damage (*p* < 0.05) (Fig. 5b). Stroke animals also experienced a decrease in both gsl and gsr compared with control animals (Fig. 5c). This deficit was largest in the contralateral forepaw (*p* < 0.01), although some reduction in grip strength was also observed in the ipsilateral forepaw (*p* < 0.05). However, the increased striatal + cortical lesion damage did not significantly worsen this deficit (Fig. 5d).

### 3.3 Correlations between ROI volume and impairment

Table 1 shows the correlations between the structural volumes and behavioural scores for the three lesion groups. The lesion volume was correlated with ffl (*r* = 0.575, *p* < 0.000001), but not ffr. The ipsilateral striatum and cortical volumes were also correlated with ffl. These trends in correlation were repeated across the lesion sub-groups but were only significant (after Bonferroni correction for multiple comparisons) in the combined group. The ipslateral ventricular volume was also correlated with ffl with a trend towards significance (*r* = 0.494, *p* = 0.0009).

The forepaw grip strength in the combined group followed a similar pattern of association, which was also repeated across the sub-groups. Note that the lesion volume (*r* = −0.392, *p* = 0.004), and the ipsilateral cortex (*r* = 0.400, *p* = 0.004) were correlated with gsl in the opposite direction to ffl (dysfunction results in higher scores for foot-faults, but lower scores for grip-strength) and approach significance after Bonferroni correction. Unexpectedly, the contralateral hippocampal volume was also weakly associated with gsl. These data indicate that the relationship between behavioural impairment and cerebral damage is complex and does not likely reflect damage to a single anatomical structure. Although correlations between lesion volumes and impairments exist, the lack of simple associations reflects the impact of stroke has on other regions. As these associations, in principle, might be detected using TBM, an association between significant TBM changes and behavioural measures could indicate the structural changes associated with outcome.

### 3.4 Voxel lesion symptom mapping

**Fig. 6:**
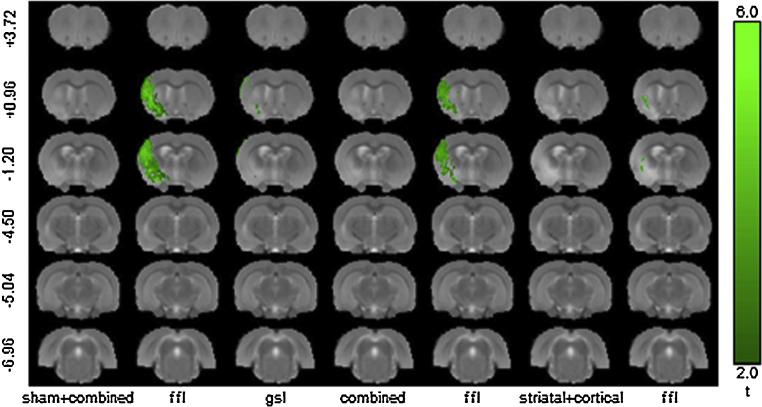
VLSM significance results. The results are shown for two behavioural tests: ffl = foot-fault-left and gsl = grip-strength-left for groups: sham + combined (all animals), combined (all stroke animals) and striatal + cortical (lesion sub-group). The slice coordinates (far left column) are relative to Bregma. In all other combinations of behaviour and lesion sub-groups, (not shown), the VLSM did not produce significant results.

The VLSM results for the combined and sham + lesion cohorts (the other lesion sub-cohorts/behaviour combinations showed no significant voxels at *q* < 0.05 or *p* < 0.01) are summarised in Fig. 6. These results are consistent with the model power analysis, which suggested that behavioural effects and samples sizes determined for ffl would be detectable in a comparable two-group problem. In addition, it is clear that MCAo (lesion), rather than filament insertion alone (sham), is the dominant effect. No significant results were detected for the ipsilateral measures (ffr and gsr).

### 3.5 Tensor-based morphometry

**Fig. 7:**
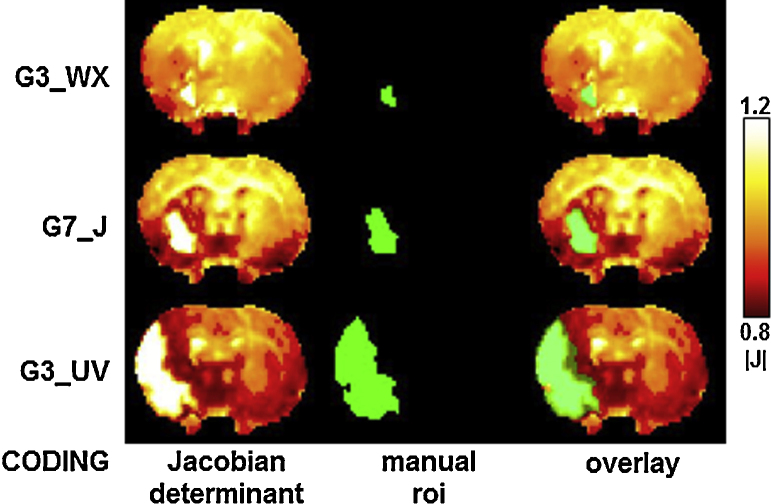
Consistency of Jacobian determinant maps derived from non-rigid registration and manually delineated lesion regions of interest. Three examples from animals with stroke lesions of varying sizes are shown. Far-left: the study coding for each animal, Left: the Jacobian determinant map derived from the non-rigid registration of each structural image to the reference; here, the lesions are shown as hyper-intense regions. Middle: the manual region of interest (drawn on the original un-registered structural images and rigidly transformed onto the reference space). Right: the manually drawn region of interest overlaid transparently in yellow on the Jacobian determinant map.

Fig. 7 shows raw Jacobian determinant maps and manual lesion delineations from three animals with varying size lesions, demonstrating that (i) hyper-intensities in the Jacobian maps are aligned closely to lesion ROIs derived from structural images and (ii) a variety of hypo- and hyper-intense features, signifying large apparent volume differences away from the lesion site, are also well resolved and clearly distinct from the lesion.

**Fig. 8:**
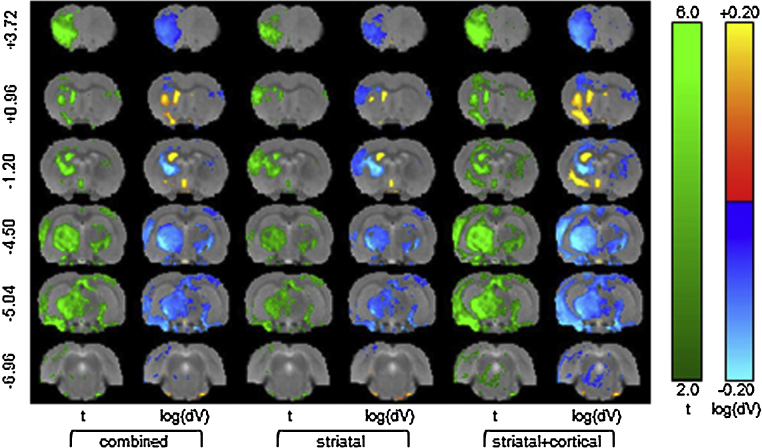
TBM results for three lesion groups compared with the sham group. In each case, the significant effects (green) are shown together with the log apparent volume differences after the FDR (*q* < 0.05) correction of multiple comparisons across voxels.

Significant TBM results are shown in Fig. 8, and the full effects and mean apparent volume change maps are shown in Fig. S1. Relative to the sham group, a small distributed volume reduction is apparent over most of the brain (Fig. S1), with a larger reduction in the ipsilateral hemisphere which is consistent with the manual segmentation results shown in Fig. 4a. The distinction between the striatal and striatal + cortical sub-groups in terms of apparent volume change in the lesioned area is clearly shown in Fig. 8. The ipsilateral ventricular enlargement remains significant after multiple-comparison corrections for all three lesion groups (combined, striatal + cortical and striatal), consistent with the largest manually measured enlargement (Fig. 4g and h). The lesioned region manifests as an apparent volume increase with a larger effect in the striatal + cortical group. The significant log-Jacobian values in Fig. 8 are in excess of the minimums for detection suggested by the model TBM power calculation.

### 3.6 Tensor-based morphometry behavioural correlation

The correlation analysis identifies regions (voxels/clusters) where behavioural scores are positively (e.g., *higher* (*lower*) score is associated with local apparent volume *increase* (*decrease*)) or negatively (e.g., *higher* (*lower*) score is associated with local volume *decrease* (*increase*)) correlated. As shown in Fig. 5, there were large variations in the behaviours between the sham and lesion groups, but the differences in the behaviours within the lesion group and between the lesion sub-groups were much smaller. We therefore applied TBM to the entire cohort (sham + combined) and the combined lesion and sub-lesion groups.

Fig. S2a–d shows the raw correlation maps of local apparent volume changes derived from fluid registration with each behavioural test for various groupings of the cohort. It is apparent that a distributed correlation structure exists with a maximum observed correlation of approximately ±0.5; this correlation is consistent with the significance/power level used in the model power calculation. In addition, this correlation structure was not confined to the lesion locale, suggesting that remote structural differences were also associated with behavioural differences. At Bregma −1.2 in the lesion vicinity, there is a clear positive correlation of *dV* (the local apparent volume difference with respect to the reference), with contralateral foot-faults (ffl) and a negative correlation of *dV* with contralateral grip-strength. This correlation is not apparent in the striatal + cortical lesion sub-group (Fig. S2d), indicating that the striatal lesion-sub-group (Fig. S2c) is responsible for this association.

**Fig. 9:**
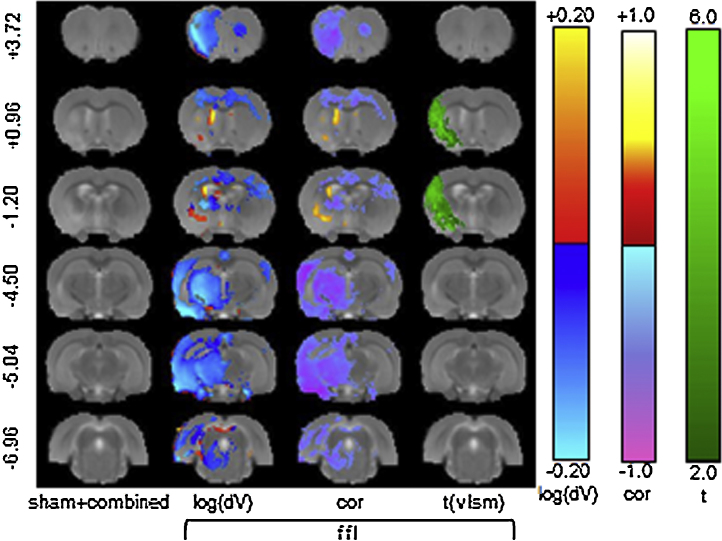
Significant TBM correlations for the contralateral foot-fault (ffl) task with apparent volume difference, and comparison with VLSM. (a) Apparent volume changes (*dV*), correlation maps (corr) and VLSM effect-sizes (*t*) are shown after correction for multiple comparisons using FDR (*q* < 0.05).

Fig. 9 shows the significant correlations and associated apparent volume differences for the sham + combined group and a comparison between the corresponding VLSM results. The ffl task produced the only significant correlations localised to the ipsilateral striatum, motor and sensorimotor cortex. However, contralateral associations with changes in the contralateral motor and sensorimotor cortex area were also evident. These results suggest that changes remote from the primary lesion are likely involved in behavioural changes. These results are consistent with the behavioural correlations observed with manual structural volume measurement shown in Table 1. Moreover, these results also show a significant gradation of structural change and behavioural dysfunction within the combined lesion group. The results shown in Fig. S2c and d suggest that these correlations are influenced through distinctions between striatal and striatal + cortical lesions; however, the results for either of these sub-groups in isolation did not reach significance.

Although these correlations provide important information concerning whether the local changes are relevant to behavioural functions, it is not clear whether these associations contribute to persisting impairment, suggesting compensatory changes, or represent indirect correlations (i.e., not causally related).

## 4 Discussion

To establish the relationship between brain damage and outcome, we must understand how a stroke lesion directly induces behavioural deficits and how subtle distributed changes also affect outcome. Advanced image analysis tools, such as voxel lesion symptom mapping (VLSM) and tensor-based morphometry (TBM), have the potential to provide a comprehensive analysis of structure and function derived from imaging and observed behaviour. In animals, these techniques will eventually facilitate the investigation of the microscopic and molecular neuro-pathological correlates of impairment relevant to morphological changes through the specific comparisons of these regions with those of control animals. As these analyses measure structural changes and do not rely on the blood oxygenation level dependent (BOLD) effect underpinning fMRI, these methods enable researchers to use complex tasks that could not be performed in a scanner (or under anaesthesia in the case of animals), while avoiding potential artefactual localisations due to angiogenesis or changes in neurovascular coupling. However, VLSM and TBM rely on a number of assumptions that, if broken, could also potentially yield artefactual results.

### 4.1 Reliability of image registration

There is insufficient information in MR images to precisely map individual voxels to another, but consistent, smooth mapping of small regions is possible, facilitating the statistical analysis of localised shape changes derived from registration. Non-rigid registration methods rely on parameters, which characterise these transformations; we use a fluid registration, which balances local displacement against local deformation, and investigated stability of the registration when varying this balance.

However, as with most voxel-based methods, non-rigid registration cannot distinguish intensity changes resulting from local structural alterations from those due to scanner variability or artefacts. Therefore, we assume that these changes, if present, are either global or random, affecting the entire cohort therefore resulting in a loss of power (by increasing local variance), rather than contributing a systematic effect to the analysis. However, abnormal hypo- or hyper-intense regions associated with lesions will also contribute to apparent local volume changes during non-rigid registration. However, these changes occur in concert with population structural variations and abnormal structural changes resulting from the lesion. Our results suggest that both types of changes can be detected and interpreted, provided that approximate knowledge of the lesion location is available. Long-term study designs, incorporating a broader imaging battery are required to understand acute structural effects (e.g., Karki et al., 2010) and their relationship to more subtle long-term effects, such as global cerebral atrophy (Walters et al., 2003).

### 4.2 Statistical power

**Fig. 10:**
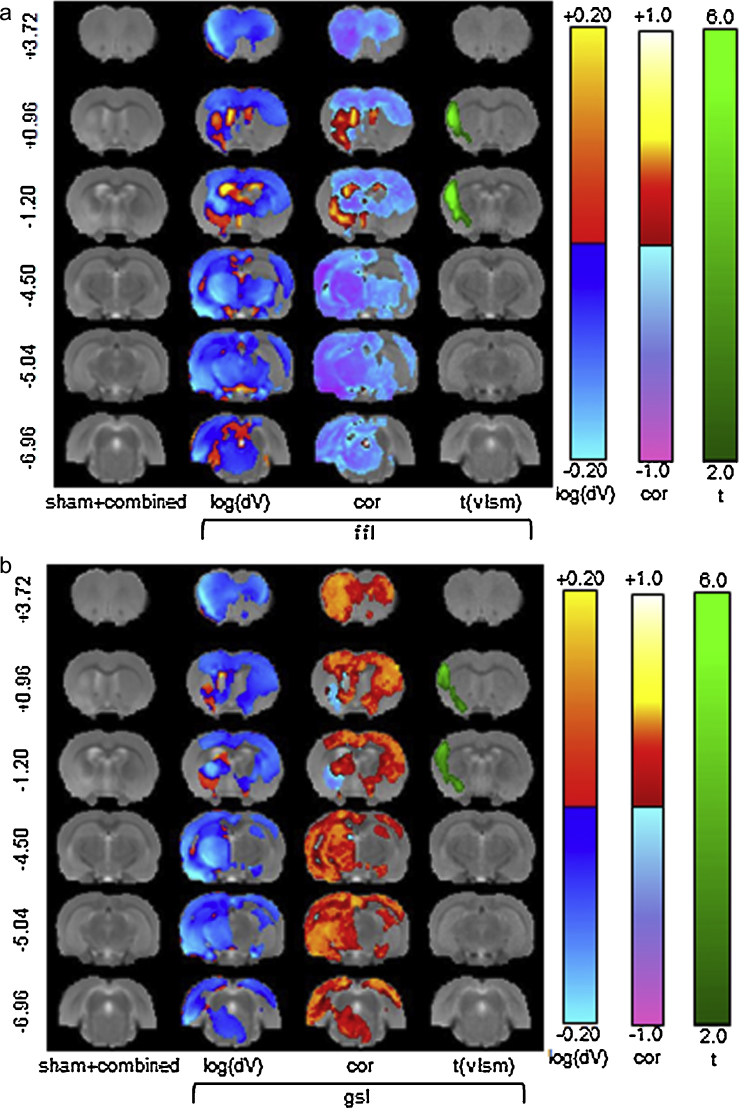
The effect of threshold free cluster enhancement (TFCE) on significance of TBM and VLSM behaviour correlation. (a) ffl and (b) gsl.

Ultimately, the success of any method based on statistics depends on the available power (i.e., the probability of correctly rejecting the null-hypothesis). In practice, power is a complicated function of the group size and the expected versus actual effect size. Therefore, the appropriateness of VLSM and TBM will be influenced by the practicalities of the scanning study, the magnitude of the structural effects and the ability of the image registration strategy to encode the effects of interest, while remaining robust to other confounding factors. Although pre-clinical studies introduce control and measurement opportunities into the scanning protocol, these studies also have practical limitations in terms of available group sizes (Suckling et al., 2010), as recently reported for the power calculations for effect size in a hypothetical multi-centre cross-sectional structural MR study. The effective power varied regionally in the brain, as a function of effect and sample sizes. To determine changes in the grey matter in sub-cortical regions of interest in psychosis studies, sample sizes of 40–80 subjects in each arm of a two-group cross-sectional study were required for 80% power, even for within-centre studies. These group-sizes are larger than many typical pre-clinical applications (including ours). The detection of comparable effect sizes might be limited through the sample size used in more conventional studies with 10–15 animals per condition, although the effect size in the case of stroke is significantly larger (30–40%) than the subtle structural changes observed after psychosis (2–5% structural change). Nevertheless, there is some evidence of these power limitations in the present work. In stroke models, large localised structural effects together with subtler delocalised effects are expected. Thus, it is possible to apply clustering techniques, such as threshold free cluster enhancement (TFCE) (Smith and Nichols, 2009), which boost power at the expense of spatial localisation when the cluster structure naturally appears in the data. For example, using TFCE enhanced the regions of significance (Fig. 10a) in the ffl behavioural correlations shown in Fig. 9 and revealed new significant regions for the gsl correlation run on the same group (Fig. 10b). The significant regions were large, due to the cluster-enhancement of TFCE and the large lesion-related effects; however, it is unclear whether TFCE adds value here. A broader study of TFCE in these contexts is warranted, but is beyond the scope of the present work.

An alternative approach is to switch from mass-univariate analysis to multivariate analysis using a machine-learning/pattern recognition strategy (Ecker et al., 2010). However, interpreting classification patterns, localising important structural foci and establishing biological plausibility from pattern recognition approaches is challenging. Recent descriptions of Bayesian VLSM that remove the need for multiple comparison corrections have shown that this method could also potentially decrease the number of subjects required for analysis (Chen and Herskovits, 2010), warranting further investigation. A more detailed discussion regarding the statistical power of VLSM in human studies is available (Kimberg et al., 2007; Rorden et al., 2007).

### 4.3 Correlating image data with behavioural measures

Stroke induces a variety of spontaneously recovering or long-term/permanent behavioural deficits (Modo et al., 2009, 2000a). It remains unclear to what degree these deficits are the direct consequence of the primary lesion compared with secondary degeneration. Although correlations between MR images and behaviour cannot confirm causal relationships, particularly when sample-sizes are limited (Henley et al., 2010; Whitwell, 2009), these analyses can suggest areas associated with impairment. In the present study, VLSM revealed how changes in the lesion extent influence behavioural performance. However, care in interpretation is required; for example, the loss of striatal tissue also induces changes in connected areas, such as the cortex. The TBM analysis is therefore more inclusive than the VLSM approach, as this technique also reveals changes outside of the primary lesion site, which might undergo secondary Wallerian degeneration (e.g., thalamus). If these secondary changes follow a similar temporal pattern to striatal atrophy, then correlations with the foot-fault score might be demonstrated, although damage to the striatum alone is sufficient to produce this deficit. It might therefore be more appropriate to consider that the *lack* of correlations, subject to the availability of sufficient statistical power, might indicate that areas are likely *not* associated with deficits, and the neurological cause of the deficit is contained within the remaining areas exhibiting correlations. However, “sufficient statistical power” might be a significant caveat for some pre-clinical studies. In this case, correlating behaviour with regional volumes as opposed to voxel-wise changes will be advantageous, but at the cost of manually defining the regions of interest and the loss of spatial localisation compared with a voxel-wise approach.

### 4.4 Biological interpretation of TBM

The interpretation of imaging analysis is affected not only by statistical and image analysis caveats but also biological assumptions. fMRI presupposes normal neurovascular coupling and a normally distributed vasculature. Similarly, TBM assumes that localised structural changes reflect localised biological changes. In grey matter, it is often implicitly assumed that changes reflect cellular or, more specifically, neuronal losses. In acute stroke however, cellular oedema could affect signal intensity without cellular loss and, in some cases, accompany an increase in cells (e.g., astrocytosis). Conversely, important structural changes could be hidden in conventional structural imaging. For example, Soria et al. (2008) demonstrated that thalamo-cortical connections are key to spontaneous behavioural recovery in an animal stroke model, but that the fibres are too few to be reliably detected on a T2-weighted structural image. Notably, white matter changes were also observed in our analysis of foot-faults (Fig. 10), demonstrating the potential to also detect changes in major fibre tracts.

The association of brain regions with behavioural changes is consistent with studies investigating forepaw impairments, such as sensorimotor perception, which indicated activity changes in the motor and somatosensory cortices (Dijkhuizen et al., 2003; Rehme et al., 2011; van Meer et al., 2012; Weber et al., 2008). However, fMRI results generally do not reveal changes in striatal areas or white matter tracts, as detected in the present study using structural MRI for the foot-fault test and grip strength tests of the impaired forepaw. These results are also fairly consistent with those observed in human patients experiencing motor deficits of the arm (Alexander et al., 2010; Lo et al., 2010). At present, however, it is unclear whether fMRI underestimates the areas involved in forepaw impairments, as this method primarily focuses on electrical stimulation, or whether TBM grossly overestimates the association between structure and behaviour. Importantly, fMRI does not reveal any changes within the lesion cavity and hence ignores the primary site of damage. The application of these complimentary techniques to serial imaging studies (Vernon et al., 2011) might be required to obtain comprehensive answers to these questions.

### 4.5 Future work

In the present study, we used standard implementations of three analysis techniques; however, many variations are possible. For localised lesions, TBM analysis can be conducted independently inside or outside a lesion to properly disentangle direct lesion effects from indirect remodelling effects. When individual lesion masks are available, TBM can be redefined as lesion-TBM, where groups are defined at each voxel based on lesion presence, similar to VLSM. Cluster enhancement techniques show promise for improving power at the cost of some spatial localisation; thus, understanding and controlling the trade-offs is required. Bayesian methods, similar to those recently applied to VLSM, might also be important in pre-clinical TBM.

### 4.6 Conclusion

The statistical analysis of the data derived from structural MR imaging of animal stroke models offers considerable insight into how the ischaemic damage in different brain regions affects behavioural performance. The increased level of detail and potential for larger-scale analysis, than is possible using manual methods, make these techniques highly desirable. However, in many animal studies, commonly applied techniques suffer from a lack of statistical power due to sample size restrictions. Careful study design at all stages of data collection, imaging and analysis is required to minimise unwanted sources of variance. In our stroke model, middle cerebral artery occlusion produced two distinct lesion sub-groups that ideally should be analysed separately (due to different lesion topology) at the cost of reduced group-size. Despite some of the challenges highlighted in this work, the continued development of image analysis tools for animal models of stroke and other psychiatric/neurological conditions is essential to provide a more robust translation between pre-clinical and clinical studies.

## Figures and Tables

**Fig. A1:**
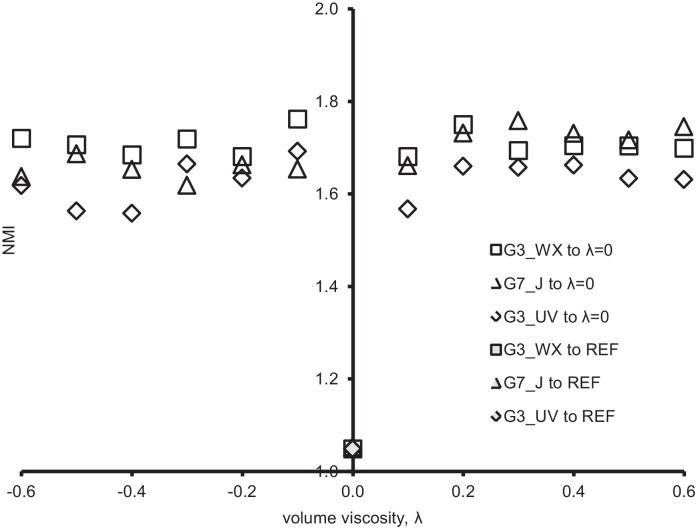
The normalised mutual information between fluid registrations with different *λ* ≠ 0 and the *λ* = 0 case. The shaded points (superimposed at the base of the *y*-axis) show the NMI between the *λ* = 0 case and the canonical reference brain.

**Fig. A2:**
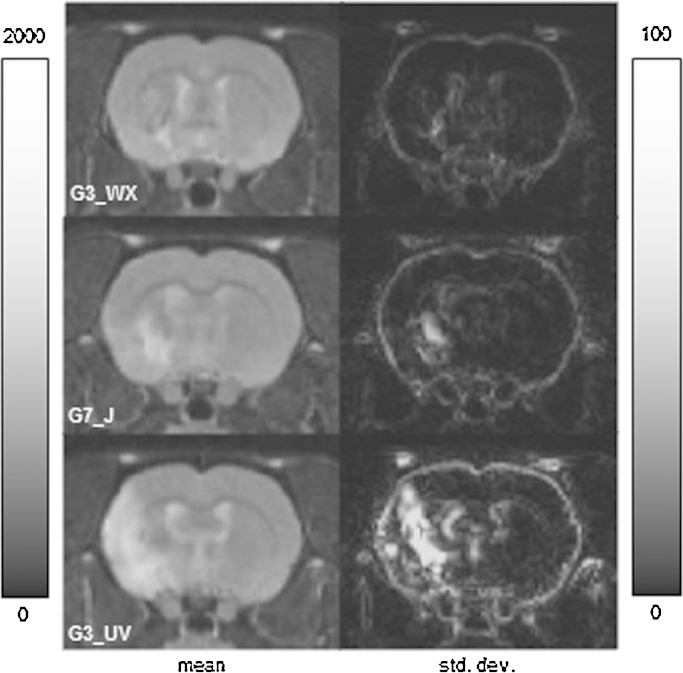
The mean and standard deviation (SD) of the registered images of the λ ≠ 0 cases. The variation in the image appearance is localised at the lesioned area and at strong boundaries. Note that the intensity scale of the SD for the images is 5% of the mean images to emphasise the variation.

**Fig. A3:**
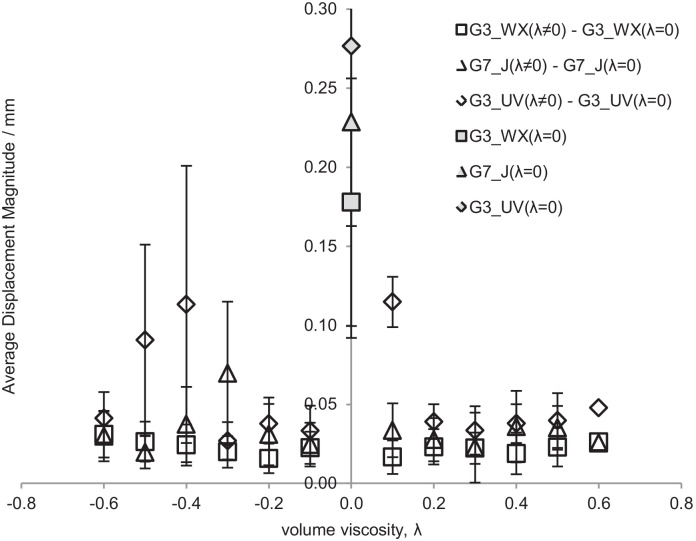
The mean difference in voxel displacement between fluid registrations with different *λ* ≠ 0 and the *λ* = 0 case. The shaded points (at the top of the *y*-axis) show the mean voxel displacement for the *λ* = 0 case registered to the canonical reference brain.

**Table 1:** Correlation coefficients between brain regions affected by stroke and behavioural impairments.

	Combined				Str.				Str + Ctx			
	Foot-faults		Grip strength		Foot-faults		Grip strength		Foot-faults		Grip strength	
	*L*	*R*	*L*	*R*	*L*	*R*	*L*	*R*	*L*	*R*	*L*	*R*
Lesion	0.575*	0.154	−0.392 (0.004)	0.079	0.422	−0.220	−0.384	0.426	0.360	−0.102	−0.322	0.125

Str												
Ipsi	−0.663**	−0.310	0.293	−0.060	−0.466	0.109	−0.550	−0.297	0.092	−0.503	−0.167	−0.327
Contra	0.222	0.018	−0.190	0.165	0.021	−0.140	−0.030	0.042	0.627	−0.002	0.280	0.468

Ctx												
Ipsi	−0.573*	−0.155	0.400 (0.004)	−0.740	−0.413	0.095	0.482	−0.235	−0.482	0.048	0.374	−0.122
Contra	−0.215	0.210	0.180	−0.036	−0.351	0.084	0.251	−0.001	−0.030	0.365	0.246	−0.073

Hipp												
Ipsi	−0.199	−0.177	0.239	0.003	−0.274	−0.320	0.382	−0.83	0.003	−0.156	0.160	0.032
Contra	−0.049	−0.117	0.393 (0.004)	0.276	−0.036	−0.370	0.368	0.493	−0.040	−0.189	0.344	−0.044

Ventr												
Ipsi	0.494 (0.0009)	0.635	−0.114	0.321	−0.199	−0.083	−0.199	0.437	0.103	0.499	0.266	0.387
Contra	0.290	−0.111	−0.83	0.221	0.351	0.031	−0.297	0.320	0.190	−0.246	0.108	0.088

Str = Striatum, Ctx = Cortex, Hipp = Hippocampus, Ventr = Ventricle, ( ), indicates *p*-values approaching significance.

* *p* < 0.000001 significant after Bonferroni correction for 108 tests.

** *p* < 0.0001 significant after Bonferroni correction for 108 tests.

## Appendix A Fluid registration parameter stability

Non-rigid registration is an essential component of tensor based morphometry analysis methods. In the present study, we use a fluid-model of registration. We emphasise that these methods are not attempting a physics-based simulation of brain tissue, but define a convenient transformation model for registration, with an excellent ability to map both highly localised and larger-scale structural differences between scans.

The fluid-model defines a class of warps from the MR image of each subject in the study to the reference scan. This class is broad, as fluid-methods successfully model large deformations and are not as constrained as methods such as those based on free form deformations defined on a grid. However, in all such methods, the warp selected from the broad class of allowed warps is governed by both the structural similarities and differences between each scan and the references, and parameters that define the fluid material properties. The Navier–Stokes equation for a compressible viscous fluid was used for the fluid registration used in the present study.

(A1) $$\mu \nabla^{2}\underline{v}+(\mu +\lambda )\nabla (\nabla \cdot \underline{v})+\underline{f}(\underline{u})=\underline{0}$$

In Eq. (A1), *v* is the instantaneous velocity of a fluid element associated with a voxel, *u* is the accrued displacement of the element to date, and *f* is an applied force driving the registration derived from local measures of image similarity. Eq. (A1) is solved for *v*, which is used to update *u*. The process is repeated to evolve *u* towards a final mapping from each scan to the reference. The parameters *μ* and *λ* are the dynamic viscosity and volume viscosity, respectively, which define the flow properties of the hypothetical fluid. Two flow mechanisms are identified in Eq. (A1) Christensen et al. (1996). Essentially, the first term (a function of *μ* only) controls the constant-volume viscous flow in the model, and the second term (a function of *μ* and *λ*) allows for local expansion or contraction. Therefore, the combined flow facilitates the displacement of misaligned structures and size/shape adjustments to accommodate the morphological differences and map regions of volume change.

The standard normalised parameter assignment in these models is *μ* = 1 and *λ* = 0, which balances the contribution of the two flow types in Eq. (A1). There is an implicit scaling of fluid material properties with the body-force, expressed as the ratio of *μ*:*λ*. In addition, material constraints require 2*μ* + 3*λ* ≥ 0. To assess the stability of the registration to parameter-choice, we ran the registration of the cases in Fig. 7 with *λ* varying in the range of −0.6 to +0.6. Therefore, *λ* < 0 reduces the impact of local volume change and *λ* > 0 increases local volume change relative to constant-volume flow (displacement).

We assessed the registration variability in two ways. First, Fig. A1, we computed the normalised mutual information (NMI) between each image output using the fluid registration with *λ* ≠ 0 and that output from the registration with *λ* = 0 to measure the effect of varying the viscosity parameters on the image similarity of the registered images to the default case. We also computed the NMI for the *λ* = 0 case and the canonical reference to measure the image similarity between an image and the reference after registration in the default case. Fig. A1 shows that varying *λ* introduces some variation in the appearance of the resulting images, but that the NMI of any *λ* ≠ 0 case with the *λ* = 0 case is much larger than that computed for different brains. Fig. A2 shows the mean and standard deviation images for the *λ* ≠ 0 cases in each of the three subjects. As expected, the variation in image appearance is largest in the lesion locale (where there is no corresponding feature in the reference) and at strong edges (where the driving forces are largest and differences in displacement versus local volume change have the largest effect).

Second, from the voxel displacement fields generated by the registration, we computed the mean *difference* in voxel displacement across the brain between the *λ* ≠ 0 and the *λ* = 0 cases and compared this value with the mean displacement of the *λ* = 0 case. Fig. A3 shows that compared with the displacements required to register each case to the reference, the variability as a function of *λ* is low and is not a strong function of *λ*. As expected, the most variation is associated with G3_UV, which had the largest lesion of the three cases studied.

## Footnotes

1 Kimberg: http://www.docstoc.com/docs/54281065/Voxel-based-mapping-of-lesion-behavior-relationships [accessed 15.05.13].

2 https://biostatistics.mdanderson.org/SoftwareDownload/SingleSoftware.aspx?Software_Id=41 [accessed 15.05.13].
