# Supplementary material for: A comparison of automated anatomical–behavioural mapping methods in a rodent model of stroke
Source: J Neurosci Methods. 2013 Sep 15;218(2):170–83. doi: 10.1016/j.jneumeth.2013.05.009 (PMC3759848; doi:10.1016/j.jneumeth.2013.05.009)
Supplement: Supplementary file 1 [file mmc1.pdf]

Supplementary Figures

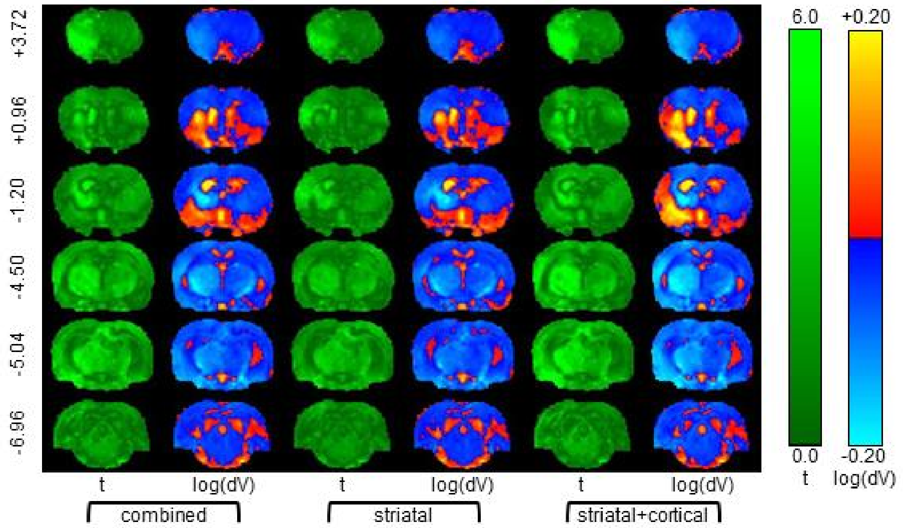

1

Supplementary Figure 1: Unthresholded TBM Results for Three Lesion Groups Compared With the Sham Group. In each case the effects (green) are shown together with log apparent volume difference. No tests for significance have been applied.

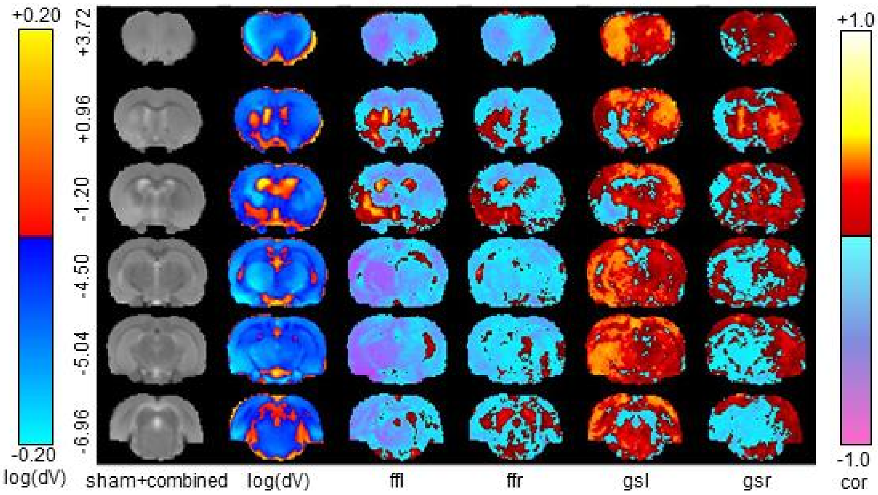
+0.20
-0.20
log(dV)
+3.72
+0.96
-1.20
-4.50
-5.04
-6.96
sham+combined
log(dV)
ffl
ffr
gsl
gsr
+1.0
-1.0
cor

2(a)

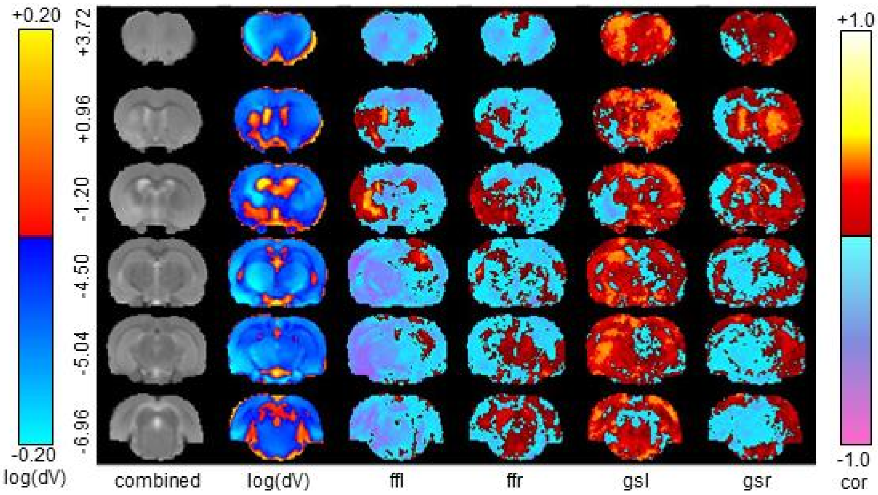
+0.20
-0.20
log(dV)
+3.72
+0.96
-1.20
-4.50
-5.04
-6.96
combined
log(dV)
ffl
ffr
gsl
gsr
+1.0
-1.0
cor

2(b)

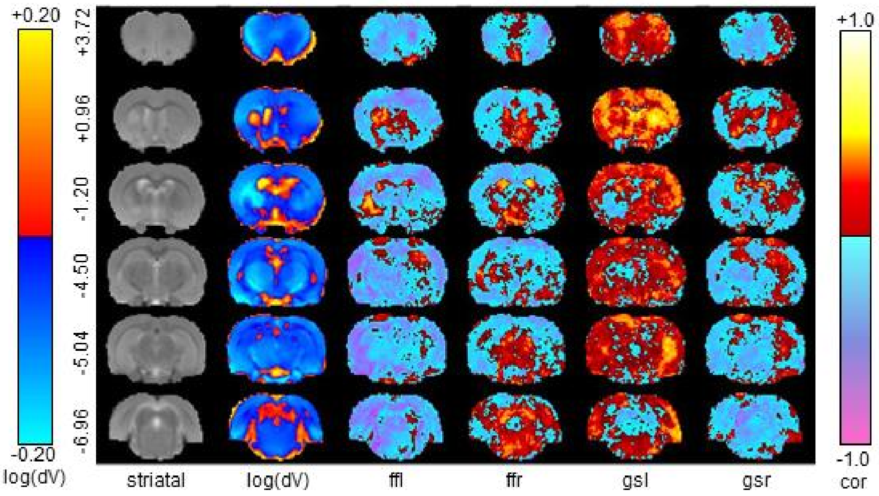
+0.20
-0.20
log(dV)
+3.72
+0.96
-1.20
-4.50
-5.04
-6.96
striatal
log(dV)
ffl
ffr
gsl
gsr
+1.0
-1.0
cor

2(c)

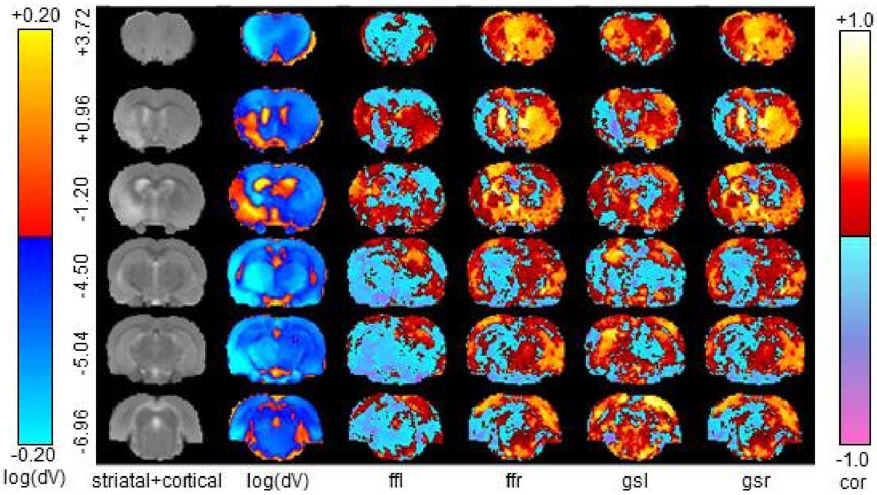

2(d)

Supplementary Figure 2: Correlation Maps of Behaviour With Volume Difference. The first column in each figure shows the mean lesion structural image in each case and the second column shows the mean volume difference (far left colour-bar) compared with the sham sub-group. The remaining four columns show the correlation maps (far right colour-bar) between the volume change and each behavioural task in turn. (a) sham+combined group, (b) combined lesion group, (c) striatal lesion sub-group, (d) striatal+cortical lesion sub-group.
